# The Relationship Between Aortic Tissue Sirtuin 1 Levels and Type A Aortic Dissections and Ascending Aortic Aneurysms

**DOI:** 10.21470/1678-9741-2024-0218

**Published:** 2025-11-17

**Authors:** Mehmet Rum, Bulend Ketenci, Fatih Kizilyel, Bahar Sarikamis Johnson, Ulkan Celik, Berfin Ekin Gozukara Yildiz, Abdulkerim Ozhan

**Affiliations:** 1 Department of Cardiovascular Surgery, Dr. Siyami Ersek Thoracic Cardiac and Vascular Surgery Hospital, University of Health Sciences, Istanbul, Turkiye; 2 Department of Medical Biology, Hamidiye Institute of Health Sciences, University of Health Sciences, Istanbul, Turkiye; 3 Department of Medical Biology, Hamidiye Faculty of Medicine, University of Health Sciences, Istanbul, Turkiye; 4 Department of Pathology, Haydarpasa Numune Education and Training Hospital, University of Health Sciences, Istanbul, Turkiye; 5 Department of Cardiovascular Surgery, Kutahya City Hospital, Kutahya Health Sciences University, Kutahya, Turkiye

**Keywords:** Aortic Aneurysm, Aortic Dissections, Ascending Aorta Aneurysm, Sirtuin 1.

## Abstract

**Introduction:**

Type A aortic dissections are pathologies with high mortality rates. Although ascending aortic aneurysms are typically planned for elective surgery, they are significant conditions in cardiovascular surgery due to their potential to cause type A aortic dissection. This study, which is the first to examine sirtuin 1 (SIRT1) in human ascending aortic tissues, aims to elucidate the relationship between ascending aortic pathologies and the SIRT1 protein.

**Methods:**

A case-control study was conducted using aortic tissues and demographic data from patients who underwent surgery for ascending aortic aneurysm and type A aortic dissection. Coronary artery bypass patients were selected as the control group. The groups were compared in terms of SIRT1 levels.

**Results:**

The study included a total of 46 patients (16 in the aneurysm group, 14 in the dissection group, and 16 in the control group). The SIRT1 protein level was the highest in the ascending aortic aneurysm group (214, interquartile range [IQR] 79 - 270), followed by the dissection group (172, IQR 148 - 224), and the lowest in the control group (104, IQR 78 - 123) (P = 0.014). SIRT1 level was found to be low in patients with coronary artery disease (P = 0.001), peripheral artery disease (P = 0.008), and hypertension (P = 0.023).

**Conclusions:**

Type A aortic dissections are associated with elevated SIRT1 levels in the tissue. Systemic atherosclerotic diseases, such as coronary and peripheral artery diseases, are associated with decreased SIRT1 levels. There is also a relationship between hypertension and sirtuin1 levels.

## INTRODUCTION

**Table:** 

Abbreviations, Acronyms & Symbols				
CAD	= Coronary artery disease		NO	= Nitric oxide
DM	= Diabetes mellitus		PAD	= Peripheral artery disease
DNA	= Deoxyribonucleic acid		SIRT1	= Sirtuin 1
eNOS	= Endothelial nitric oxide synthetase		TAA	= Thoracic aortic aneurysm
HT	= Hypertension		TBST	= Tris Buffered Saline with Tween™ 20
IQR	= Interquartile range			

Sirtuins are epigenetic regulatory proteins consisting of 200 - 275 amino acids. They are derived from the silent information regulatory 2 gene, initially discovered in yeast. Sirtuins are categorized into seven subtypes, with SIRT1, SIRT6, and SIRT7 located in the nucleus, SIRT3, SIRT4, and SIRT5 in the mitochondria, and SIRT2 functioning in the cytosol^[1]^. Sirtuin 1 (SIRT1), the most extensively studied sirtuin, plays various roles in cell aging, metabolism regulation, deoxyribonucleic acid (DNA) repair, cardiovascular protection, inflammation, apoptosis, and autophagy^[2]^ Several studies have established its association with diabetes, demonstrating increased glucose tolerance and insulin secretion upon SIRT1 overexpression^[3]^.

Indeed, there is evidence supporting the protective properties of SIRT1 against vascular diseases, which can be attributed to its antioxidant, anti-aging, anti-inflammatory, and anti-apoptotic effects on endothelial smooth muscle and adventitial tissues. SIRT1 plays a role in regulating endothelial nitric oxide synthetase (eNOS), leading to the production of nitric oxide (NO). Additionally, there is a positive feedback loop where NO increases the expression of SIRT1. It's worth noting that one mechanism of action of statins and cilostazol involves inhibiting the eNOS inhibitor N-nitro-L-arginine methyl ester (or L-NAME). These interactions highlight the intricate relationship between SIRT1, NO, and the mechanisms of action of certain medications used in vascular diseases^[4]^.

SIRT1’s protective effect against vascular diseases is attributed to its antioxidant, anti-aging, anti-inflammatory, and anti-apoptotic properties^[5]^. The main aim of the study is to investigate the relationship of SIRT1 in ascending aortic aneurysms and type 1 dissections.

## METHODS

### Design and Duration

This case-control study included patients who underwent surgery at the Dr. Siyami Ersek Thoracic Cardiac and Vascular Surgery Training and Research Hospital within a 10-month period from February 10, 2022, to December 10, 2022.The study was approved by the Clinical Research Ethics Committee of T.C. Ministry of Health Istanbul Haydarpaşa Numune Training and Research Hospital on 07.02.2022 (number: HNEAH- KAEK 2022/28-3469). The principles of the Helsinki Declaration were adhered to at every stage of the research.

### Study Population

The study was designed based on three groups, consisting of two case groups and one control group. The first case group comprises the aneurysm group, which consisted of patients who underwent surgery for ascending aortic aneurysm; the second case group comprises the dissection group, which consisted of patients who underwent surgery for type 1 and type 2 aortic dissection. Due to ethical reasons, we were unable to obtain healthy human aortic tissue for the control group in this study, which constitutes the most significant potential bias. Instead, aortic tissue from individuals with aortas within normal limits but exposed to a systemic disease such as atherosclerosis was utilized. The control group consisted of patients who underwent coronary artery bypass surgery.

### Excluded Patients

Patients under the age of 18 years, patients who have previously undergone cardiac surgery, and patients with only intramural hematoma were excluded from the study. Additionally, individuals who declined to participate were not included.

The sample consisted of 17 patients in the aneurysm group, with one patient being excluded due to accompanying type 3 dissection. Additionally, 15 patients were included in the dissection group, with one patient's tissue transfer being excluded as it was unsuitable. In the control group, samples from two out of 18 patients were excluded as they were not suitable for the study. These tissue samples were excluded due to the concern that they may introduce bias, as they contained atherosclerotic plaques.

### Tissue Collection, Transfer, and Storage

The patients who were planned to have their tissues collected and met the eligibility criteria for the study were informed before the surgery. Informed consent forms were signed by the patients themselves and their relatives if available.

During the surgical procedure, aortic tissues were excised from patients diagnosed with ascending aortic aneurysm and aortic dissection. The excised aortic tissue was separated from the adventitial tissue. After that, the remaining segment (tunica media and tunica intima) was carefully placed into a tube and subsequently transferred to a deep freezer maintained at a temperature of -80°C, using dry ice as a refrigerant for long-term storage.

### Protein Isolation and Blotting

The frozen tissue samples were taken on dry ice and cut into small pieces using a scalpel. Each patient's sample was weighed with a precision balance and then homogenized using a tissue homogenizer device with lysis buffer containing 1% protease phosphatase inhibitor at 50 Hz/min vibration. The homogenized samples were centrifuged at 14,000 g for 15 minutes at +4°C, and the resulting supernatant was collected in separate tubes. Protein concentration was measured using a spectrophotometer. Blots were prepared by mixing the total protein amount (20 - 40 µg) with lithium dodecyl sulfate and reducing agent at calculated ratios. The prepared blots were denatured at 70°C and stored at -20°C until the next experiment.

### Western Blot

**Fig. 1:**
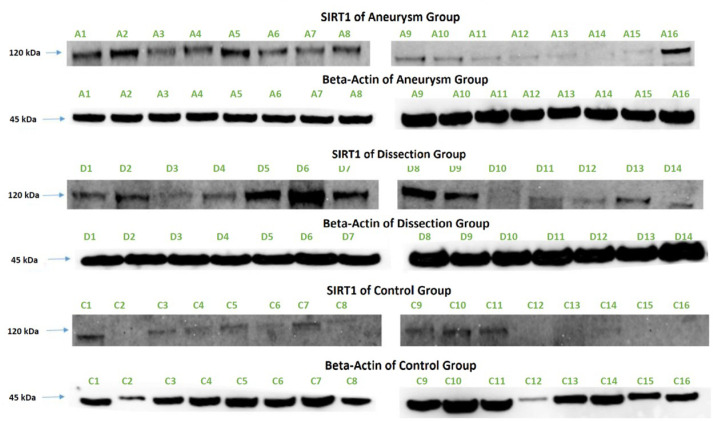
Sirtuin 1 (SIRT1) Western Blot image.

The prepared blots (20 - 40 µg) were loaded into a 4 - 12% Bolt™ gel in the tank with antioxidant and running buffer, and electrophoresis was performed for three to four hours. Then, transfer to an iBlot™ 2 polyvinylidene difluoride membrane was carried out. Blocking was performed for one to two hours using 0.1% Tris Buffered Saline with Tween™ 20 (TBST) buffer containing 5% skimmed milk powder. The prepared primary antibodies (SIRT1 and beta-actin) were incubated with the blots overnight at +4°C. The next day, after washing with TBST buffer, the blots were incubated with the appropriate concentrations of secondary antibody for one hour on a shaker. After washing following the application of the secondary antibody, images were captured using the iBright™ FL1000 device and the WesternBright™ Sirius™ Chemiluminescence Imaging Kit (Figure 1).

### Variables

The patient’s aortic diameters were obtained from preoperative computed tomography or echocardiograms. Ejection fractions were derived from preoperative echocardiograms. Data concerning comorbidities (peripheral artery disease [PAD], hypertension [HT], coronary artery disease [CAD], diabetes mellitus [DM]) were evaluated based on the patients' previous hospital visits, diagnostic tests, medications, and medical histories. Additionally, PADs were assessed based on concurrently conducted computed tomographies and angiographic interventions. Information regarding body surface area, age, and sex was retrieved from patient records.

### Statistical Methods

The statistical analysis was conducted using the R statistical package (R version 4.2.2, Vienna, Austria). Categorical data were presented as numbers and percentages, normally distributed continuous data as mean and standard deviation, and non-normally distributed continuous data as median and quartiles. The comparison between groups was performed using Chi-squared test, *t*-test, and Mann-Whitney U test. For the comparison of three groups, Chi-squared test, analysis of variance, and Kruskal-Wallis test were used. Spearman’s correlation analysis was used to assess the correlation between continuous variables. A significance level of *P* < 0.05 was considered statistically significant. While evaluating PADs, clear data for two patients could not be obtained. These are missing data. These data have been excluded from the evaluation and calculations.

## RESULTS

The study included a total of 46 patients, with 13 (28.2%) being female and 33 (71.8%) being male. The age range of the participants was 19 to 78 years. The median age for all groups combined was 64 years, while the aneurysm group had a median age of 66 years (interquartile range [IQR], 48 - 71), the dissection group had a median age of 55 years (IQR, 51 - 69), and the control group had a median age of 65 years (IQR, 59 - 68).

Comparison of median body surface areas revealed that the control group had a value of 1.85 m^2^ (1.75 - 1.89), the aneurysm group had a value of 1.95 m^2^ (1.78 - 2.02), and the dissection group had a value of 2.06 m^2^, indicating a significant difference between the groups (Table 1).

**Table 1:** Demographics and laboratory findings.

	Aneurysm^1^	Dissection^1^	Control^1^	*P*-value
	(n = 16)	(n = 14)	(n = 16)	
Male (n = 33)	12	9	12	0.8^2^
	-75%	-64%	-75%	
Female (n = 13)	4	5	4	
	-25%	-36%	-25%	
Age (years)	66	55	65	0.6^3^
	(IQR, 48 - 71)	(IQR, 51 - 59)	(IQR, 59 - 68)	
Body surface area (m^2^)	1.95	2.06	1.85	0.049^3^
	(IQR, 1.78-2.02)	(IQR, 1.87 - 2.13)	(IQR 1.75 - 1.89)	
Aortic diameter (mm)	53	49	35	0.001^3^
	(IQR, 52 - 58)	(IQR, 44 - 57)	(IQR, 32 - 36)	
Diabetes mellitus (n = 15)	12.5%	14.2%	68.75%	0.001^2^
	(n = 2)	(n = 2)	(n = 11)	
Hypertension (n = 22)	44%	57%	44%	0.6^4^
	(n = 7)	(n = 8)	(n = 7)	
Coronary artery disease (n = 24)	44%	7.1%	100%	0.001^4^
	(n = 7)	(n = 1)	(n = 16)	
Peripheral artery disease (n = 7)	12.5%	0%	31%	0.010^2^
	(n = 2)	(n = 0)	(n = 5)	
SIRT1	214	172	104	0.014^3^
	(IQR, 79 - 270)	(IQR, 148 - 224)	(IQR, 78 - 123)	

1 n (%); median (IQR);

2 Fisher's exact test;

3 Kruskal-Wallis rank sum test;

4 Pearson's Chi-squared test

IQR=interquartile range; SIRT1=sirtuin 1

It was observed that in the sample group, diabetes was present in 33% of the patients, HT in 48%, and PAD in 16%. In terms of diabetes, patients with DM comprised 12.5% (n = 2) of the aneurysm group, 14.2 % (n = 2) of the dissection group, and 68.75% (n = 11) of the control group. PAD was observed in seven patients, with two in the aneurysm group and five in the control group. PAD status of two patients in the dissection group was not clearly known (Table 1).

**Fig. 2:**
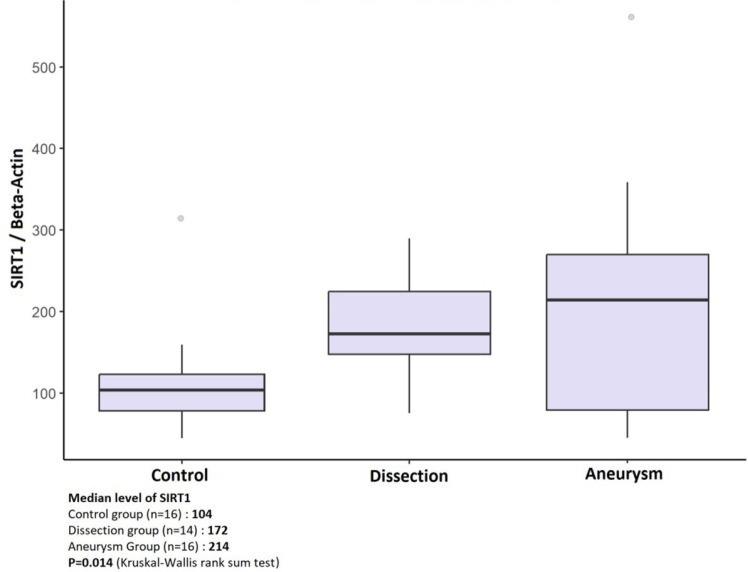
Sirtuin 1 (SIRT1) distribution diagram by groups.

The median SIRT1 value was found to be the highest in the aneurysm group at 214 (IQR 79 - 270); intermediate in the dissection group at 172 (IQR 148 - 224); and the lowest in the control group at 104 (IQR 78 - 123). The difference between the groups was statistically significant (*P* = 0.014) (Figure 2). The difference between these groups lies in the comparison between the dissection group and the control group (Table 2).

**Table 2:** The significance of sirtuin 1 (SIRT1) differences according to binary groups.

	SIRT1	(*P*-value)^*^
Aneurysm // control	214 (IQR, 79 - 270) // 104 (IQR, 78 - 123)	0.08
Dissection // control	172 (IQR, 148 - 224) // 104 (IQR, 78 - 123)	0.001
Aneurysm // dissection	214 (IQR, 79 - 270) // 172 (IQR, 148 - 224)	> 0.09

* Wilcoxon rank sum exact

Note: aneurysm group n = 16; dissection group n = 14; control group n = 16

IQR=interquartile range

In the overall patient population, when dividing them into two groups based on the presence (n = 24) or absence (n = 22) of CAD, the SIRT1 levels were calculated as 93 (75 - 123) in patients with CAD and 216 (150 - 246) in the group without CAD. The SIRT1 level was higher in aortic tissue in patients with CAD (*P* < 0.001) (Table 3).

**Table 3:** Statistical results of sirtuin 1 (SIRT1) with hypertension (HT), coronary artery disease (CAD), and peripheral artery disease (PAD).

	Patients	SIRT1^a^	*P*-value^*^
CAD	CAD+ (n = 24)	93 (IQR, 75 - 123)	< 0.001
	CAD- (n = 22)	216 (IQR, 150 - 246)	
PAD	PAD+ (n = 7)	79 (IQR, 63 - 108)	= 0.008
	PAD- (n = 37)	149 (IQR, 106 - 223)	
HT	HT+ (n = 22)	118 (IQR, 75 - 148)	= 0.023
	HT- (n = 24)	184 (IQR, 111 - 244)	

* Wilcoxon rank sum exact test;

a Median (IQR)

IQR=interquartile range

**Fig. 3:**
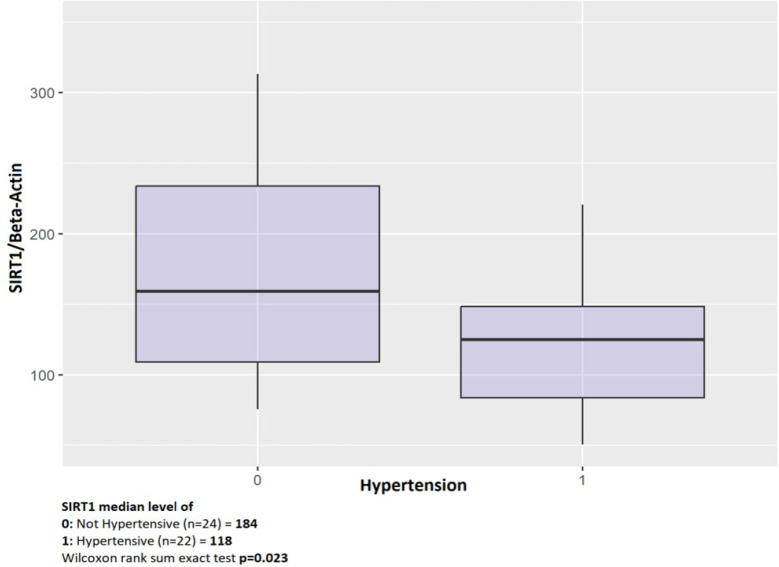
Diagram of hypertension with sirtuin 1 (SIRT1).

The patient group (n = 7) with PAD had a significantly lower SIRT1 level compared to the group without PAD (n = 37) (*P* = 0.008) (Table 3). When examining the patients in two groups based on the presence (n = 22) or absence (n = 24) of HT, the SIRT1 level was lower and statistically significant in the group with HT compared to the group without HT (*P* = 0.023) (Figure 3). However, there was no significant difference in the SIRT1 level between patients with (n = 15) and without (n = 31) DM.

**Fig. 4:**
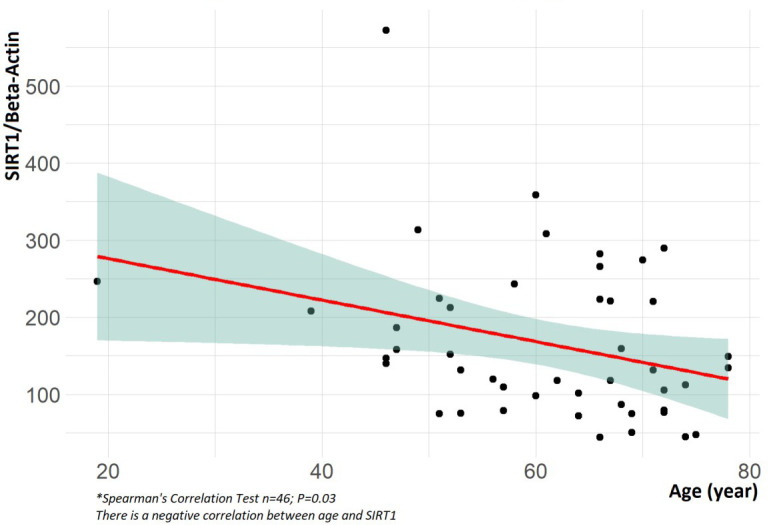
Correlation between sirtuin 1 (SIRT1) and age.

**Fig. 5:**
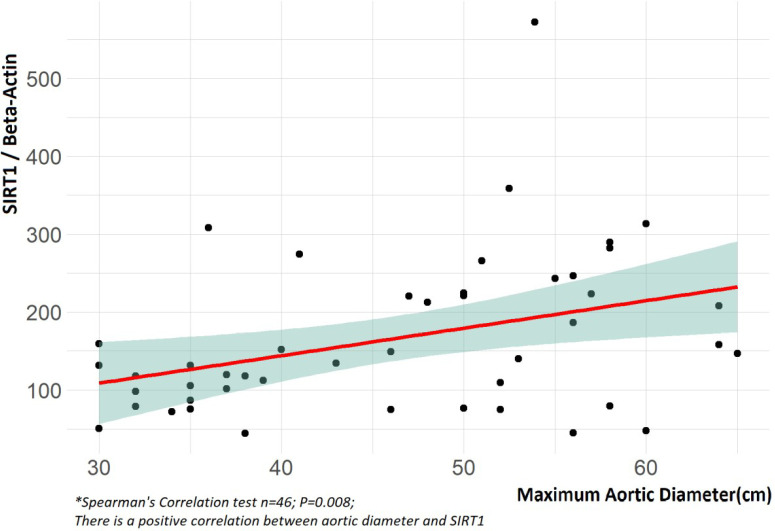
Correlation between sirtuin 1 (SIRT1) and maximum aortic diameter.

The SIRT1 values were found to be negatively correlated with age (*P* = 0.03, *Ρ* < 0.032), meaning that as age increases, SIRT1 levels decrease (Figure 4). On the other hand, there was a positive correlation between SIRT1 values and aortic diameter (*P* = 0.008, *Ρ* = 0.385), indicating that as the aortic diameter increases, the level of SIRT1 also increases (Figure 5) (Table 4).

**Table 4:** Correlation of sirtuin 1 (SIRT1) with age and aortic diameter.

SIRT1	ρ	*P*-value^*^
Age	- 0.320	0.03
Aortic diameter	0.385	0.008

* Spearman correlation test

Note: when calculating the correlation between age, aortic diameter, tunica media, and intima with SIRT, all participants in the study population were included, n = 46

## DISCUSSION

This study aimed to establish a SIRT1 database in human aortic tissues to assess the potential utility of SIRT1 protein as a biomarker and evaluate its role in aortic aneurysms and dissections. The elevation of SIRT1, which is considered a vascular protective protein, was observed to be associated with the ascending aortic dissection group, contrary to the literature. Considering that the control group consisted of coronary artery bypass patients affected by systemic atherosclerosis, SIRT1 levels were observed to be low in vascular aging and atherosclerosis, consistent with the literature.

In Fang Wang's study, they administered a chemical that induces thoracic aortic aneurysm (TAA) and aortic dissection in mice and compared mice with high and low levels of smooth muscle-specific SIRT1. The study reported that the strain with the highest smooth muscle-specific SIRT1 had lower mortality^[6]^. However, the findings from our study showed different results. In our study, when comparing SIRT1 levels in the aortic tissues of the three groups (aneurysm, dissection, and control), the highest level was observed in the aneurysm group (214), followed by the dissection group (172), and the lowest level was in the control group (104) (*P* = 0.014). This contrasts with the notion that SIRT1 elevation is protective against ascending aortic aneurysms and aortic dissection, as suggested in the literature.

Examining TAA under two groups, distal and proximal to the ligamentum arteriosum, it has been observed that atherosclerosis plays a role in the pathogenesis of aneurysms distal to the ligament, while aneurysms proximal to the ligament are not atherosclerotic^[7]^. The ascending aorta, with greater longitudinal tension and elasticity compared to the descending aorta, is believed to have different smooth muscle cell origins^[8]^. Smooth muscle cells in the aorta proximal to the ligamentum arteriosum originate from the neural crest, while those distal to it come from the paraxial mesoderm^[9-14]^. Experimental studies did not specify which segments of the TAA develop aneurysms. 3-aminopropionitrile fumarate (or BAPN) is a compound that has been used in experimental studies to induce aortic aneurysms in mice. It acts as a lysyl oxidase inhibitor^[15]^, affecting collagen production. Collagen is an important component of the extracellular matrix in blood vessels. SIRT1 has been shown to increase the production of type I collagen from fibroblasts^[16]^.

However, it is important to note that in TAA, the pathology is not solely characterized by underproduction of collagen. There are multiple factors and processes involved in the development and progression of TAAs, including inflammation, oxidative stress, genetic predispositions, and alterations in extracellular matrix components.

SIRT1 plays a role in the production of eNOS in endothelial cells. NO, produced by eNOS, is an antioxidant chemical responsible for vascular relaxation and proliferation. SIRT1 and NOS have a mutualistic relationship with positive feedback. In our study, when patients were grouped as hypertensive (n = 22) and non-hypertensive (n = 24), SIRT1 levels were higher in the non-hypertensive group. This mutualistic relationship between SIRT1 and NOS, which promotes vascular relaxation, may contribute to the protective effect of SIRT1 against HT^[17,18]^.

In an experimental study conducted by Rateri et al., it was demonstrated that angiotensin II contributes to the development of ascending aortic aneurysm, while SIRT1 inhibits its harmful effects in aneurysm tissue^[19]^. When comparing patients with and without HT, it was found that hypertensive patients were associated with lower SIRT1 levels, consistent with the literature. However, contrary to this example, our study demonstrated that patients with aortic dissection had higher SIRT1 levels compared to the control group. No significant difference in SIRT1 levels was observed between the aneurysm group and the control group.

In Fry et al.'s experimental study^[20]^, exogenous angiotensin II was infused into the aortic walls of normal mice with and without SIRT1 destruction. The results showed that mortality due to aortic dissection, particularly in the thoracic region, increased by 70% in mice with SIRT1 destruction. This was attributed to the involvement of SIRT1 in the aortic wall in oxidant and inflammatory stimulation. SIRT1 has an anti-inflammatory effect by suppressing the production of matrix metalloproteinase.

It's important to consider these contrasting findings and the complexity of the mechanisms involved in aortic pathologies. Further research is needed to better understand the role of SIRT1 in the development of TAA and aortic dissections, taking into account factors such as collagen production, atherosclerosis, and the specific segments of the aorta involved.

Studies using human tissues, such as the internal mammary artery, aorta, and carotid endarterectomy samples, have demonstrated the presence of SIRT1, which plays a role in preventing DNA damage and may have a protective effect against atherosclerosis. However, the study mentioned that suggests SIRT1's potential regression of medial degeneration was based on mouse experiments, not human tissue^[21]^. In ascending aortic aneurysms and aortic dissections, the main pathology involves medial degeneration rather than atherosclerosis. We believe that our study, being the first investigation conducted on human ascending aortic tissues, will make a valuable contribution to the literature. While our study yielded results consistent with the literature regarding the role of SIRT1 in atherosclerosis-related diseases, further studies conducted on a broader range of human tissues are needed to evaluate its role in pathologies characterized by medial degeneration, such as ascending aortic aneurysms and type 1 dissections.

When the sample group was divided into two groups based on the presence (n = 24) or absence (n = 22) of CAD, it was observed that among the patients who underwent open-heart surgery, those with CAD were younger than those without CAD (*P* = 0.012). Additionally, SIRT1 levels were found to be lower in the group without CAD (93) compared to the group with CAD (216) (*P* = 0.001). Similarly, SIRT1 levels were lower in the group without PAD (n = 37) compared to the group with PAD (n = 7) (*P* = 0.008). In our study, it was also observed that the low levels of atherosclerosis-based vascular diseases are associated in parallel with the literature^[22]^.

Cell senescence is a condition where cells deteriorate and lose their ability to reproduce. There are two types of cell senescence. Replicative cell senescence occurs naturally when cells are unable to reproduce due to the shortening of telomeres^[23]^. Over time, cells reach a limit known as the Hayflick limit^[24]^, after which replication ceases. Stress-induced premature senescence occurs when cell proliferation stops prematurely due to factors such as oxidative stress or DNA damage. Vascular aging refers to the aging of endothelial cells in the intima layer and vascular smooth muscle cells in the media layer of blood vessel walls. Senescent cells undergo morphological and physiological changes, leading to inflammation, atherosclerosis, and thrombosis in vascular cells, as well as problems with vascular relaxation, angiogenesis, and regeneration^[22,24-27]^.

Sirtuins are a group of proteins with nicotine adenine dinucleotide-dependent deacetylation or adenosine diphosphate ribosyl transferase activity. In endothelial cells, SIRT1 inhibits p53 deacetylation and hydrogen peroxide production. Hydrogen peroxide is a chemical that causes oxidative stress and contributes to premature cell aging^[5,28]^. In our study, we observed a negative correlation (*P* = 0.03) between the SIRT1 results and the ages of the cases, which provides valuable insights that can contribute to the existing literature.

### Limitations

During the design phase of the study, the intention was to obtain non-aneurysmal aortic tissue as control samples, adhering to ethical guidelines. However, due to limitations and availability of suitable control tissue, punch samples taken during proximal anastomosis in coronary artery bypass surgery were used as a substitute. It is acknowledged that the control group having CAD may not have been an ideal reference for comparing with aneurysmal and dissected aortic tissues.

It is important to note that the availability of appropriate control tissue can sometimes be challenging in research studies, and researchers often need to make practical considerations when selecting control groups. While the inclusion of CAD in the control group may introduce some confounding factors, it is still valuable to compare and analyze the available data to gain insights and generate hypotheses for further investigations.

Future studies may benefit from obtaining dedicated non-aneurysmal aortic tissues as controls or considering alternative approaches to minimize potential confounders, thus providing a more accurate comparison between aneurysmal, dissected, and non-diseased aortic tissues.

## CONCLUSION

In this study conducted on human aortic tissues, it was observed that low SIRT1 levels were associated with diseases developing on the basis of atherosclerosis (CAD, PAD), as well as HT, consistent with the literature. A negative correlation was observed between age and SIRT1 levels.

Contrary to the literature, it was found that high SIRT1 levels were associated with type A aortic dissections.

By acknowledging the need for larger studies, we highlight the importance of further investigation to confirm and expand upon our findings. Continued research in this area will enhance our understanding of SIRT1’s role in various cardiovascular conditions and its potential implications for clinical practice.

## Data Availability

The authors declare that due to patient confidentiality, the data have not been publicly shared; however, they can be provided in an anonymized and encrypted format upon request to the authors.

## Artificial Intelligence Usage

The authors declare use of Grammarly OpenAI for minor grammar checking and minor editing. The content produced by the artificial intelligence tool was revised and edited by the authors as necessary, and they take full responsibility for the content to be published.

## References

[1] Wang F, Chen HZ. (2020). Histone deacetylase SIRT1, smooth muscle cell function, and
vascular diseases. Front Pharmacol.

[2] Ulu İ, Çakmak GG, Çelik Sk (2021). Sirtuin 1 ve sirtuin 2’nin tip 2 diyabet ile
ilişkisi. Turk J Diab Obes.

[3] Moynihan KA, Grimm AA, Plueger MM, Bernal-Mizrachi E, Ford E, Cras-Méneur C (2005). Increased dosage of mammalian Sir2 in pancreatic beta cells
enhances glucose-stimulated insulin secretion in mice. Cell Metab.

[4] Budbazar E, Rodriguez F, Sanchez JM, Seta F. (2020). The role of sirtuin-1 in the vasculature: focus on aortic
aneurysm. Front Physiol.

[5] Kida Y, Goligorsky MS. (2016). Sirtuins, cell senescence, and vascular aging. Can J Cardiol.

[6] Wang F, Tu Y, Gao Y, Chen H, Liu J, Zheng J. (2020). Smooth muscle sirtuin 1 blocks thoracic aortic
aneurysm/dissection development in mice. Cardiovasc Drugs Ther.

[7] Elefteriades JA, Farkas EA. (2010). Thoracic aortic aneurysm clinically pertinent controversies and
uncertainties. J Am Coll Cardiol.

[8] de Beaufort HW, Nauta FJ, Conti M, Cellitti E, Trentin C, Faggiano E (2017). Extensibility and distensibility of the thoracic aorta in
patients with aneurysm. Eur J Vasc Endovasc Surg.

[9] Serra R, Grande R, Montemurro R, Butrico L, Caliò FG, Mastrangelo D (2015). The role of matrix metalloproteinases and neutrophil
gelatinase-associated lipocalin in central and peripheral arterial
aneurysms. Surgery.

[10] Huusko T, Salonurmi T, Taskinen P, Liinamaa J, Juvonen T, Pääkkö P (2013). Elevated messenger RNA expression and plasma protein levels of
osteopontin and matrix metalloproteinase types 2 and 9 in patients with
ascending aortic aneurysms. J Thorac Cardiovasc Surg.

[11] Jackson V, Olsson T, Kurtovic S, Folkersen L, Paloschi V, Wågsäter D (2012). Matrix metalloproteinase 14 and 19 expression is associated with
thoracic aortic aneurysms. J Thorac Cardiovasc Surg.

[12] Saeyeldin AA, Velasquez CA, Mahmood SUB, Brownstein AJ, Zafar MA, Ziganshin BA (2019). Thoracic aortic aneurysm: unlocking the "silent killer"
secrets. Gen Thorac Cardiovasc Surg.

[13] Cheung C, Bernardo AS, Trotter MW, Pedersen RA, Sinha S. (2012). Generation of human vascular smooth muscle subtypes provides
insight into embryological origin-dependent disease
susceptibility. Nat Biotechnol.

[14] Tadros TM, Klein MD, Shapira OM. (2009). Ascending aortic dilatation associated with bicuspid aortic
valve: pathophysiology, molecular biology, and clinical
implications. Circulation.

[15] Sigma-Aldrich 3-Aminopropionitrile fumarate salt, metabolite.

[16] Yang Y, Li Z, Guo J, Xu Y. (2020). Deacetylation of MRTF-A by SIRT1 defies senescence induced
down-regulation of collagen type I in fibroblast cells. Biochim Biophys Acta Mol Basis Dis.

[17] Tanno M, Sakamoto J, Miura T, Shimamoto K, Horio Y. (2007). Nucleocytoplasmic shuttling of the NAD+-dependent histone
deacetylase SIRT1. J Biol Chem.

[18] Mattagajasingh I, Kim CS, Naqvi A, Yamamori T, Hoffman TA, Jung SB (2007). SIRT1 promotes endothelium-dependent vascular relaxation by
activating endothelial nitric oxide synthase. Proc Natl Acad Sci U S A.

[19] Rateri DL, Moorleghen JJ, Balakrishnan A, Owens AP, Howatt DA, Subramanian V (2011). Endothelial cell-specific deficiency of Ang II type 1a receptors
attenuates Ang II-induced ascending aortic aneurysms in LDL receptor-/-
mice. Circ Res.

[20] Fry JL, Shiraishi Y, Turcotte R, Yu X, Gao YZ, Akiki R (2015). Vascular smooth muscle sirtuin-1 protects against aortic
dissection during angiotensin ii-induced hypertension. J Am Heart Assoc.

[21] Marampon F, Gravina GL, Scarsella L, Festuccia C, Lovat F, Ciccarelli C (2013). Angiotensin-converting-enzyme inhibition counteracts angiotensin
II-mediated endothelial cell dysfunction by modulating the p38/SirT1
axis. J Hypertens.

[22] Gorenne I, Kavurma M, Scott S, Bennett M. (2006). Vascular smooth muscle cell senescence in
atherosclerosis. Cardiovasc Res.

[23] Collado M, Blasco MA, Serrano M. (2007). Cellular senescence in cancer and aging. Cell.

[24] Hayflick L, Moorhead PS. (1961). The serial cultivation of human diploid cell
strains. Exp Cell Res.

[25] van Deursen JM (2014). The role of senescent cells in ageing. Nature.

[26] Erusalimsky JD. (2009). Vascular endothelial senescence: from mechanisms to
pathophysiology. J Appl Physiol (1985).

[27] Chen J, Goligorsky MS. (2006). Premature senescence of endothelial cells: Methusaleh's
dilemma. Am J Physiol Heart Circ Physiol.

[28] Ota H, Akishita M, Eto M, Iijima K, Kaneki M, Ouchi Y. (2007). Sirt1 modulates premature senescence-like phenotype in human
endothelial cells. J Mol Cell Cardiol.

